# Predictors of micronutrient powder (MNP) knowledge, coverage, and consumption during the scale‐up of an integrated infant and young child feeding (IYCF‐MNP) programme in Nepal

**DOI:** 10.1111/mcn.12712

**Published:** 2019-10-17

**Authors:** Lindsey M. Locks, Pradiumna Dahal, Rajkumar Pokharel, Nira Joshi, Naveen Paudyal, Ralph D. Whitehead, Stanley Chitekwe, Zuguo Mei, Bikash Lamichhane, Aashima Garg, Maria Elena Jefferds

**Affiliations:** ^1^ Department of Nutrition Harvard T.H. Chan School of Public Health Boston Massachusetts USA; ^2^ Nutrition Section United Nations Children's Fund (UNICEF) Headquarters New York New York USA; ^3^ Nutrition Section United Nations Children's Fund (UNICEF) Kathmandu Nepal; ^4^ Nutrition Section Government of Nepal Ministry of Health and Population Kathmandu Nepal; ^5^ New ERA Kathmandu Nepal; ^6^ Nutrition Branch, Division of Nutrition, Physical Activity, and Obesity United States Centers of Disease Control and Prevention Atlanta Georgia USA; ^7^ Child Health Division Government of Nepal Ministry of Health and Population Kathmandu Nepal

**Keywords:** adherence, anaemia, coverage, implementation, infant and young child feeding (IYCF), micronutrient powders (MNP)

## Abstract

Large‐scale programmes using micronutrient powders (MNPs) may not achieve maximum impact due to limited/inappropriate MNP coverage, consumption, and use. We identify predictors of MNP coverage, maternal knowledge of appropriate use, and child MNP consumption in Nepal. A cross‐sectional survey was conducted in 2,578 mother–child pairs representative of children 6–23 months in two districts that were part of the post‐pilot, scale‐up of an integrated infant and young child feeding‐MNP (IYCF‐MNP) programme. Children aged 6–23 months were expected to receive 60 MNP sachets every 6 months from a female community health volunteer (FCHV) or health centre. Outcomes of interest were MNP coverage (ever received), maternal knowledge of appropriate use (correct response to seven questions), repeat coverage (receipt ≥ twice; among children 12–23 months who had received MNP at least once, *n* = 1342), and high intake (child consumed ≥75% of last distribution, excluding those with recent receipt/insufficient time to use 75% at recommended one‐sachet‐per‐day dose, *n* = 1422). Multivariable log‐binomial regression models were used to identify predictors of the four outcomes. Coverage, knowledge of appropriate use, and repeat coverage were 61.3%, 33.5%, and 45.9%, respectively. Among MNP receivers, 97.9% consumed MNP at least once and 38.9% of eligible children consumed ≥75% of last distribution. FCHV IYCF‐MNP counselling was positively associated with knowledge, coverage, repeat coverage, and high intake; health worker counselling with knowledge and coverage indicators; and radio messages with coverage indicators only. FCHV counselling had the strongest association with knowledge, coverage, and high intake. Community‐based counselling may play a vital role in improving coverage and intake in MNP programmes.

**Key messages**

- Large‐scale programmes distributing MNP may have less impact in reducing anaemia and iron deficiency than efficacy trials due to reduced coverage and adherence; however, few large‐scale programmes have representative indicators of programme implementation.
- Collecting quantitative programme implementation data in a sample that is representative of a large programme area can allow programme implementers and evaluators to assess which programme indicators (i.e., coverage, knowledge, and adherence) are successfully being achieved and also enable them to identify sociodemographic and programme‐related variables that can predict these outcomes. In Nepal, hearing MNP radio messages and health worker and FCHV counselling were independently associated with MNP coverage; however, FCHV counselling had the strongest association with coverage, maternal knowledge of appropriate use, and high child intake of MNP.
- Community‐based counselling may play a key role in improving coverage and intake in MNP programmes globally; future research should address how to utilize community‐health workers without over‐burdening their workload.
- Continued resources and support for MNP programmes as they scale to the national and subnational level during post‐pilot phase are essential to support programme coverage and the adoption and maintenance of positive behaviour change.

## 1 INTRODUCTION

Undernutrition during the first 1,000 days—from conception to 2 years—increases the risk of illness, death, and developmental delays in childhood (Black et al., 2013). Micronutrient deficiencies are particularly prevalent—globally, 43% of children aged 6–59 months in low‐ and middle‐income countries suffer from anaemia, approximately a quarter of which is due to iron deficiency (Petry et al., 2016). The World Health Organization (WHO) recommends the introduction of diverse, solid and semi‐solid, and complementary foods at age 6 months because breastmilk is no longer sufficient to meet children's nutritional needs during this critical time period (Brown, Dewey, & Allen, 1998; WHO, 2003). In resource‐limited settings, however, micronutrient‐rich foods such as animal‐source foods are often inaccessible for families, and when they are available, they often are not provided in sufficient quantities to young children (Dewey & Adu‐Afarwuah, 2008; Murphy & Allen, 2003). Currently, only one in six children aged 6–23 months in low‐ and middle‐income countries are fed the minimum acceptable diet (defined as minimum meal frequency of solid or semi‐solid foods and minimum dietary diversity; UNICEF, 2016).

Micronutrient powders (MNPs)—which come in single‐dose, light‐weight, and shelf‐stable sachets—can be mixed with a variety of semi‐solid foods to increase the availability of vitamins and minerals in children's diets (Zlotkin et al., 2005). Meta‐analyses of randomized trials have found that regular MNP consumption can reduce the risk of anaemia and iron deficiency in children aged 6–23 months by approximately 25% and 50%, respectively (De‐Regil, Suchdev, Vist, Walleser, & Peña‐Rosas, 2013; Salam, MacPhail, Das, & Bhutta, 2013); however, interventions using MNP implemented outside of controlled trials have had smaller impacts (Hirve et al., 2013; Locks et al., 2017; Serdula et al., 2013; Vossenaar et al., 2017).

Many countries are implementing programmes using MNP in communities where complementary feeding practices are suboptimal and micronutrient deficiencies are common. MNPs are often used instead of iron drops/syrups due to research showing MNPs have higher acceptability, fewer side effects, and similar efficacy (Dewey, Yang, & Boy, 2009). From 2011 to 2015, the number of countries implementing programmes with MNP tripled from 22 to 65, with over 10 million children aged 6–59 months receiving MNP in 2015 (UNICEF, 2017). Of the 65 countries implementing programmes with MNP in 2015, 25 had initiated national or subnational programmes (UNICEF, 2017). Most programmes distribute MNP free of charge through the health sector, usually as part of ongoing infant and young child feeding (IYCF) programmes (Reerink et al., 2017). The free health sector MNP programmes, which usually distribute MNP through routine health facility visits, health facility visits and community outreach, or biannual child health days, have coverage rates ranging from 32% to 83% based on each programme's definition of coverage (Jefferds et al., 2015; Korenromp et al., 2016; Reerink et al., 2017), and intake adherence (defined by each programme) of 35–88% (Jefferds et al., 2015; Korenromp et al., 2016; Reerink et al., 2017; World Vision, 2005). Notably, the majority of evaluations reporting coverage and intake data have come from pilot interventions in small areas with more resources than would be available during national or subnational implementation (Jefferds et al., 2015; Korenromp et al., 2016; Reerink et al., 2017; World Vision, 2005). A recent MNP expert consultative group concluded that implementation research from programmes operating at scale (defined as reaching a large population with long‐term delivery infrastructure) is urgently needed to inform MNP programmes globally (Nyhus Dhillon et al., 2017).

In this paper, we analyse household survey data representative of children aged 6–23 years and their mothers in two districts that were part of the post‐pilot, scale‐up of the integrated IYCF‐MNP programme in Nepal. We identify demographic characteristics, indicators of programme exposure (such as exposure to mass media, health worker, and/or community health worker counselling), and maternal and child experiences with MNP that predict key MNP programme indicators of coverage, repeat coverage, knowledge of appropriate use, and adherence.

## 2 METHODS

### 2.1 Study population and data collection

MNP has been locally branded in Nepal as *Baal Vita* (translated as “Vitamins for Children”); each sachet contains 15 micronutrients including iron and zinc at ~1x the Recommended Nutrient Intake (WFP/WHO/UNICEF, 2007). After a feasibility study assessed the acceptability of MNP and developed key messages and strategies for the integrated IYCF‐MNP programme, a pilot programme was conducted in 2010–2011 where MNP was delivered in some districts through government‐run health facilities and in other districts through female community health volunteers (FCHVs; Jefferds et al., 2015). Beginning in late 2012, the Ministry of Health and the United Nations Children's Fund (UNICEF) began scaling‐up the integrated IYCF‐MNP programme, which as of 2016 had reached 26 out of the country's 75 districts. The integrated programme includes IYCF‐MNP counselling and the distribution of MNP by both health workers and FCHVs. The analyses here are from a cross‐sectional “endline” survey conducted 3 years after the IYCF‐MNP programme had been operational in two of the scale‐up districts (Kapilvastu in the plains and Achham in the hills ecological zones).

Details on programme implementation from the Nepal scale‐up of the integrated IYCF‐MNP programme have been published (Locks et al., 2018). In brief, a cascade training approach using the UNICEF community‐based IYCF training tools (UNICEF, 2012), with additional modules on MNP, adapted for the Nepali context. Health workers and FCHVs were trained on key components of IYCF such as the promotion of exclusive breastfeeding for the first 6 months, timely introduction of complementary foods, and the importance of diverse and frequent complementary feeding, as well as on the appropriate distribution, storage, and use of MNP. Both health workers and FCHVs were expected to counsel caregivers on IYCF and MNP in order to utilize multiple contact points with consistent messages (our study found that over three quarters of mothers had interacted with both their FCHV and health worker about the health of her child). Health workers were expected to counsel mothers during routine health visits, and FCHVs were to hold monthly mothers group meetings, where they would counsel mothers using the IYCF‐MNP counselling cards and flipcharts. In accordance with the national FCHV programme, FCHVs, who volunteer their time, were also encouraged to conduct home visits or individual counselling if/when they had the time to do so. The current WHO guidelines recommend three boxes (90 sachets) of MNP over a 6‐month period (WHO, 2016); however, the Nepal IYCF‐MNP programme, which was rolled out before the newest WHO guidelines, recommended that mothers receive 60 sachets of MNP every 6 months from an FCHV or health worker (whomever she preferred/interacted with first); mothers were then instructed to feed their child one sachet/day, with an expected 4‐month gap after finishing the 60 sachets before the next distribution.

As part of the programme scale‐up, MNP were first distributed by FCHVs and health workers in Achham and Kapilvastu in March 2013; however, subsequent distributions were interrupted due to issues with the international supplier which resulted in a temporary national stock‐out. Achham and Kapilvastu did not have the expected second distribution of 60 sachets in 2013. A partial distribution using MNP from the emergency stock (noncustomized general brand) occurred in January 2014 with a *Baal Vita* sticker (similar to that of customized brand) on the box; the IYCF components of the programme continued during MNP stock disruptions. New customized *Baal Vita* arrived in country in March 2014, and refresher trainings and a relaunch of the integrated IYCF‐MNP intervention were carried out. Also in 2014, another project started also supporting general IYCF practices in Kapilvastu and Achham, but the programme did not distribute, provide training on, or promote MNP (Cunningham et al., 2017). Full MNP distributions relaunched in Kapilvastu in May 2014 and Achham in June 2014, providing sufficient time for the oldest children in the survey (aged 18–23 months) to complete all three expected distribution cycles (60 sachets every 6 months) before the end line survey.

The district‐representative survey was conducted among children aged 6–23 months and their mothers in Kapilvastu and Achham in January–February 2016. Population proportional to size sampling was used to select 40 clusters from each district. A census of young children in selected clusters was conducted to identify all children aged 6–23 months; random sampling was used to select 34 children from each cluster in Kapilvastu and 33 in Achham. There was no replacement for refusals or clusters with less than the needed number of children. Trained interviewers used a structured questionnaire, the majority of questions had pretested during the pilot intervention. Mothers were asked about demographic characteristics and experiences with the integrated IYCF‐MNP programme. Specifically, mothers were asked how often they sought help/counselling for their child from an FCHV or health worker, whether they discussed child feeding with either provider, the amount of time spent travelling to each (by foot, bike, motorcycle, car, or bus) and satisfaction level with the services (very satisfied, satisfied, not satisfied, or very unsatisfied). If the mother reported discussing child feeding with her FCHV, she was asked whether this occurred individually, in a group, or both. Mothers were also asked if they had heard of *Baal Vita* (MNP), and if so, where did they heard about it. They were also asked seven knowledge questions on appropriate use: at what age the child should begin MNP, at what age MNP was no longer necessary, how many sachets a child should consume per day, what size portion of food MNP should be added to, whether MNP should be added to food while cooking or hot, whether MNP should be added to liquids, and within what timeframe after adding MNP food should be consumed. Mothers were also asked whether they had ever received MNP for the child, and if so, how many times; when, where, and how many sachets they last received at their last distribution; and whether they received a reminder to pick up MNP before their last distribution (and if so from whom). All questions on MNP receipt and intake were based on maternal recall, though notably, a study from the Nepal pilot IYCF‐MNP programme found that maternal recall on the number of sachets consumed and the number of sachets observed were highly correlated (Ng'eno et al., 2017). Mothers who had never received MNP were asked why not; mothers who had received MNP were asked if they had ever fed their child MNP. Mothers who had fed their child MNP were asked whether they identified changes in the colour, taste, or smell of the food, and if so, whether those changes bothered the child. They were also asked how many sachets the child had consumed from the last batch, whether the child liked food with MNP, and whether they noticed any positive or negative changes in their child since initiating MNP.

### 2.2 Ethics

Ethical approval was obtained from the Nepal Health Research Council (NHRC). Survey enumerators described the purpose, procedures, risks, and benefits of the study and allowed mothers to ask questions before inviting them to participate in the survey. All mothers provided written informed consent to enrol themselves and their children in the survey; if mothers were illiterate, a witness signature was obtained.

### 2.3 Data analysis

Descriptive statistics are presented as frequencies and percentages; the average for the two districts was weighted based on district population. Binary variables were created for four outcomes of interest. Our selection of programmatic outcomes of interest was guided by the WHO/CDC logic model for micronutrient interventions in public health programmes (De‐Regil, Pena‐Rosas, Flores‐Ayala, & del Socorro Jefferds, 2014). At the household level, improving micronutrient status (in this case through the consumption of MNP) is dependent on the target population using the intervention appropriately, which has two proximate inputs: (a) intervention coverage and (b) a target population that knows, demands, accepts, and has the ability to appropriately use the intervention (De‐Regil et al., 2014). A prerequisite for MNP intake is thus access to or presence of MNP sachet stock in the community via FCHV or from health facilities. It has also been proposed that caregiver adherence with MNP recommendations can be conceptualized as containing three key elements: initiation, appropriate use, and continued use of MNP (Tumilowicz, Schnefke, Neufeld, & Pelto, 2017). Our four outcomes of interest were thus MNP coverage (ever received MNP), repeat MNP coverage (receipt of MNP ≥2 times among mothers of children aged 12–23 months who had received MNP at least once), maternal knowledge of appropriate use (based on seven knowledge questions), and high child intake adherence (consumption of ≥75% of last MNP distribution). There is no consensus on how to define intake adherence for MNP programmes (Reerink et al., 2017). In Nepal, mothers were expected to receive two boxes (60 sachets) of MNP during each distribution; however, many reported receiving only one box (30 sachets) at the last distribution. We defined “high intake” as consumption of ≥75% of the last batch of MNP received (among mothers who received MNP) accounting for when MNP was received; mothers who did not have sufficient time to use ≥75% of their sachets at the recommended dose of one sachet per day were removed from the analyses (i.e., mothers who received 30 sachets within the last 23 days or 60 sachets in the last 45 days). In order to confirm that it was appropriate to collapse mothers who received 60 and 30 sachets into a single group with a cut‐off of 75% for high intake, we used a chi‐square test to confirm there was not a significant difference in the proportion of mothers who fed their child ≥75% of the MNP received based on whether the mother received one or two boxes at the last distribution. Because almost all mothers in the survey who received MNP initiated use (fed the child MNP at least once), this is not included as an independent indicator.

Chi‐squared tests were used to assess whether demographic characteristics were associated with each of the four outcomes of interest. Demographic characteristics considered were district, child's sex, child's age, ethnicity, maternal education, source of household income, household food insecurity based on the Household Food Insecurity Access Scale (Coates, Swindale, & Bilinsky, 2007), and household asset tertile (developed from a principle component analysis based on household ownership of electricity, radio, television, mobile, refrigerator, table, chair, bed, sofa, watch, computer, fan, traditional grain miller, and bicycle). All demographic characteristics associated (*p* < 0.20) with each outcome in chi‐squared tests were included in all multivariable models for that outcome. Multivariable generalized estimating equations (GEE) using a log link, binomial distribution, and exchangeable correlation structure to account for clustering were used to estimate prevalence ratios for each outcome. This model was selected over logistic regression because logistic regression odds ratios would have overestimated the prevalence ratio for the association between exposures and outcomes given the high prevalence of many of our outcomes of interest (Spiegelman & Hertzmark, 2005). When the log‐binomial model did not converge (as indicated in the footnotes in Table 4), a Poisson distribution was used because it gives a consistent estimate of the relative risk (Zou, 2004).

Multivariable log‐binomial and log‐Poisson models with exchangeable correlation structures were also used to estimate prevalence ratios for each outcome of interest with programme exposure indicators as exposures. Programme exposure indicators included amount of time mother spent travelling to her health centre and FCHV, exposure to sources of MNP information (radio messages, health worker counselling, and FCHV counselling), the type of FCHV IYCF‐MNP counselling (individual, group, both, or neither), whether the mother received a reminder to pick‐up the last distribution from an FCHV or health worker, and specific FCHV and health centre indicators such as frequency of interactions and maternal satisfaction with the services provided (due to the low prevalence of mothers who were unsatisfied with their FCHV, we compared “very satisfied” versus “satisfied, unsatisfied, or very unsatisfied”). We also assessed whether maternal experiences with and perceptions of MNP were predictors of mothers receiving MNP a second time (repeat coverage) and high child intake of MNP. For models with programme exposure indicators or maternal perceptions as exposures, the same demographic characteristics associated with each outcome (*p* < 0.20) in chi‐squared tests were included in each model. Determination of which additional programme indicators were included as covariates in the multivariable models were determined a priori based on directed acyclic graphs of how programme indicators were hypothesized to influence each other (see Table S1). All analyses were conducted in SAS 9.4 (Cary, NC).

## 3 RESULTS

A total of 2,578 mother–child pairs participated in the survey, representing 96% of those invited. Approximately half of the children were male, and one third fell in each age category (6–11, 12–17, and 18–23 months**;** Table 1 **)**. Three quarters of mothers had sought help/counselling for the child aged 6–23 months from both a health centre and an FCHV, and only 2% of mothers had never interacted with either.

**Table 1:** Sociodemographic characteristics and access to health services among mothers and children aged 6–23 months in Kapilvastu and Achham districts, Nepal

	**Kapilvastu** a	**Achham** a	**Weighted Total** a
	***n* = 1,345**	***n* = 1,233**	***n* = 2,578**
**Sociodemographic characteristics**			
Child's age			
6–11 months	402 (29.9)	418 (33.9)	808 (31.3)
12–17 months	519 (38.6)	448 (36.3)	974 (37.8)
18–23 months	424 (31.5)	367 (29.8)	796 (30.9)
Child's sex			
Male	718 (53.4)	651 (52.8)	1370 (53.2)
Female	627 (46.6)	582 (47.2)	1207 (46.8)
Ethnicity			
Upper caste	189 (14.1)	840 (68.1)	869 (33.7)
Dalit hill/Terai	220 (16.4)	379 (30.8)	557 (21.6)
Other	936 (69.6)	14 (1.1)	1152 (44.7)
Maternal education			
No formal education	623 (46.4)	663 (53.8)	1263 (49.1)
Primary education	280 (20.8)	151 (12.3)	457 (17.7)
Secondary education or higher	441 (32.8)	418 (33.9)	856 (33.2)
Number of people sharing a kitchen			
Tertile 1 (≤5 people)	418 (31.1)	568 (46.1)	941 (36.5)
Tertile 2 (6–7 people)	322 (23.9)	371 (30.1)	675 (26.2)
Tertile 3 (≥8 people)	605 (45.0)	292 (23.8)	962 (37.3)
Main source of household income			
Agriculture	769 (57.2)	737 (59.8)	1498 (58.1)
Remittance	117 (13.7)	259 (22.6)	413 (16.0)
Other	342 (26.6)	151 (13.2)	667 (25.9)
Household food security level (Coates et al., 2007)			
Food secure	908 (67.5)	486 (39.4)	1477 (57.3)
Mildly food insecure	106 (7.9)	145 (11.8)	240 (9.3)
Moderately food insecure	207 (15.4)	248 (20.1)	441 (17.1)
Severely food insecure	124 (9.2)	354 (28.7)	420 (16.3)
Household assets Tertile^b^			
Tertile 1 (more assets)	543 (40.4)	374 (30.3)	947 (36.7)
Tertile 2	607 (45.1)	379 (30.7)	1028 (39.9)
Tertile 3 (fewer assets)	195 (14.5)	480 (38.9)	603 (23.4)
**Maternal experience with health centres and female community health volunteers (FCHVs)**			
Mother has sought help/counselling about the health of her child from:			
Both her FCHV and a government health centre	970 (72.1)	1082 (87.8)	2005 (77.8)
A government health centre only	110 (8.2)	64 (5.2)	183 (7.1)
Her FCHV only	219 (16.3)	78 (6.3)	327 (12.7)
Neither her FCHV or a government health centre	46 (3.4)	9 (0.7)	63 (2.4)
Frequency of health facility visits focused on the child			
Less than once per month	790 (58.7)	459 (37.2)	1312 (50.9)
Monthly	344 (25.6)	376 (30.5)	706 (27.4)
More than once per month	211 (15.7)	298 (32.3)	560 (21.7)
Amount of time it takes to travel to the health centre^c^			
≤30 min	798 (73.9)	553 (48.3)	1393 (63.7)
>30 min	282 (26.1)	593 (51.8)	795 (36.3)
Mother reports discussing the child's feeding with a health worker	728 (54.1)	903 (73.2)	1574 (61.1)
Relating to the quality of care received at the health centre, mother reports being:^c^			
Very satisfied	146 (13.5)	190 (16.6)	322 (14.7)
Satisfied	894 (82.8)	942 (82.2)	1806 (82.5)
Not satisfied, very unsatisfied or no response	40 (3.7)	14 (1.2)	59 (2.7)
Frequency of mother‐FCHV interactions focused on the child			
Less than once per month	523 (38.9)	315 (25.6)	877 (34.0)
Monthly	403 (30.0)	358 (29.0)	763 (29.6)
More than once per month	354 (26.3)	203 (16.5)	936 (36.3)
Amount of time it takes to travel to meet the FCHV^d^:			
Mother only received home visits	281 (24.2)	169 (14.6)	471 (20.5)
Mother travels ≤20 min	795 (68.4)	731 (63.1)	1525 (66.4)
Mother travels >20 min	87 (7.5)	258 (22.3)	302 (13.1)
Mother reports discussing the child's feeding with the FCHV	991 (73.7)	1030 (83.5)	1991 (77.3)
Relating to the quality of care provided by the FCHV, mother reports being^d^:			
Very satisfied	213 (17.9)	228 (19.7)	433 (18.6)
Satisfied	942 (79.2)	919 (79.2)	1847 (79.2)
Not satisfied or very unsatisfied	34 (2.9)	13 (1.1)	51 (2.2)

a All values are frequency *n* (%).

b Household assets tertile determined from principle component analysis that included questions on whether each household had a radio, television, mobile phone, refrigerator, table, chair, bed, sofa, watch, computer, fan, dhiki, bicycle, and electricity.

c Only among mothers who have sought help/counselling from a health centre (*n* = 1,080 in Kapilvastu and *n* = 1,146 in Achham). Mothers indicated amount of time based on their primary mode of transportation, which could be walking, bicycle/rickshaw, or motorbike/vehicle/bus.

d Only among mothers who have sought help/counselling from an FCHV (*n* = 1,189 in Kapilvastu and *n* = 1,160 in Achham). Mothers indicated amount of time based on their primary mode of transportation, which could be walking, bicycle/rickshaw, or motorbike/vehicle/bus.

Approximately half of mothers of children 6–23 months in Kapilvastu and three‐quarters in Achham had received MNP at least once; however, only one‐quarter and one‐half of mothers of children aged 12–23 months in each district respectively had received MNP at least twice **(**Table 2 **)**. Among mothers of children 12–23 months who had received MNP once, approximately half (40.4% in Kapilvastu and 53.9% in Achham) received a second MNP distribution (the definition of “repeat coverage”). Among mothers in both districts, one third correctly answered all seven appropriate use questions with correct responses to individual questions ranging from 54.4% to 78.5%. Among mothers who received MNP (with sufficient time to feed the child one sachet/day and use three quarters of the sachets), 38.9% had fed their child ≥75% of the sachets received. Among mothers who tried giving their child MNP, approximately half thought the child liked food with MNP, whereas half reported that their child was disturbed by a change of colour, taste, or smell of food. In exploratory chi‐squared analyses, we did not find an association between maternal knowledge of appropriate use of MNP and maternal report of organoleptic changes (changes in taste, smell, or colour) of food after adding MNP. Specifically, mothers who correctly answered all seven appropriate use questions were just as likely to identify organoleptic changes in food after adding MNP than mother who did not correctly answer all seven questions (48.3% vs. 43.9%, *p* = 0.18). We also did not find any differences among mothers who specifically knew that MNP should not be added to hot foods (63.5% vs. 64.9%, *p* = 0.68) or liquids (64.4% vs. 60.8%, *p* = 0.26). Half of mothers who tried MNP reported positive effects such as improvements in their child's health, growth, immunity, appetite, energy/activity, or mental development, whereas one third reported negative effects such as black or loose stool, constipation, vomiting, or nausea.

**Table 2:** Indicators relating to micronutrient powder (MNP) knowledge, coverage, intake, and perceptions among mothers of children 6–23 months in Kapilvastu and Achham districts in Nepal

	**Kapilvastu** a	**Achham** a	**Weighted Total** a
	***n* = 1,345**	***n* = 1,233**	***n* = 2,578**
**Maternal knowledge of appropriate use of MNP** b			
Mother knows MNP should be introduced at 6 months	809 (60.2)	962 (78.0)	1717 (66.6)
Mother knows that MNP are no longer necessary after 23 months	675 (50.2)	763 (61.9)	1403 (54.4)
Mother knows that one sachet per day is the recommended dose	930 (69.1)	996 (80.8)	1891 (73.4)
Mother knows that MNP should be added to a small portion of food	995 (74.0)	1064 (86.3)	2022 (78.5)
Mother knows that MNP should not be added to hot food	822 (61.1)	884 (71.7)	1674 (65.0)
Mother knows that MNP should not be added to liquids	765 (56.9)	935 (75.8)	1643 (63.8)
Mother knows that food with MNP should be consumed within 30 min of adding MNP	986 (73.3)	1072 (86.9)	2058 (78.3)
Mother knows correct answer to at least 6 of the 7 knowledge questions above	694 (51.6)	821 (66.6)	1470 (57.1)
Mother knows correct answer to all 7 questions above (knowledge of appropriate use indicator)	400 (30.0)	498 (40.4)	866 (33.6)
**MNP coverage and intake**			
Mother reports that she has received MNP for the child at least once (coverage indicator)	732 (54.4)	903 (73.2)	1579 (61.3)
Among mothers of infants 12–23 months^c^, mother received MNP ≥ two times	256 (27.2)	382 (46.9)	602 (34.0)
Among mothers who had received MNP once and whose children were 12–23 months^d^, mother received MNP ≥ two times (repeat coverage indicator)	256 (40.4)	382 (53.9)	602 (45.9)
Among mothers who received MNP^e^, timing and quantity of the last distribution was:			
The recommended 60 sachets during the last 6 months	254 (35.2)	339 (37.9)	567 (36.4)
Sixty sachets more than 6 months ago	45 (6.2)	43 (4.8)	88 (5.6)
A different quantity of sachets in the last 6 months^f^	343 (47.5)	427 (47.8)	743 (47.6)
A different quantity of sachets more than 6 months ago^f^	80 (11.1)	85 (9.5)	162 (10.4)
Among mothers who received MNP^e^, mother identifies her female community health volunteer (FCHV) as her primary source of MNP	640 (87.6)	744 (82.7)	1346 (85.4)
Among mothers who received MNP^e^, mother identifies a health centre as primary MNP source	74 (10.1)	140 (15.6)	197 (12.5)
Among mothers who received MNP^e^, mother fed her child MNP at least once	718 (98.2)	877 (97.6)	1542 (97.9)
Among mothers who received MNP^g^, child consumed ≥75% of sachets received from last batch of MNP sachets received (high intake indicator)	274 (42.3)	266 (34.4)	536 (38.9)
Among mothers who never received MNP^h^, reasons cited include:			
Mother did not know she was supposed to get MNP for her child	276 (45.0)	130 (39.4)	435 (43.6)
Stock‐out at the FCHV or health facility	113 (18.4)	67 (20.3)	189 (18.9)
Mother did not want to use MNP^i^	228 (37.2)	104 (31.5)	357 (35.8)
External barriers^j^	7 (1.1)	12 (3.6)	18 (1.8)
**MNP experiences and perceptions among mothers who ever fed MNP to her child** k			
Mother reports that the child likes to eat food with MNP	385 (53.6)	433 (49.2)	798 (51.7)
Mother reports that adding MNP changed the colour of the food	232 (32.3)	323 (36.7)	528 (34.2)
Mother reports that adding MNP changed the taste of the food	320 (44.5)	439 (49.9)	724 (46.8)
Mother reports that adding MNP changed the smell of the food	173 (24.1)	253 (28.8)	403 (26.1)
Mother reports that the child disliked the change in colour, taste or smell of the food	320 (44.5)	459 (52.2)	739 (47.8)
Mother perceived a positive effect of MNP in the child^l^	357 (49.7)	460 (52.3)	785 (50.8)
Mother perceived a negative effect of MNP in the child^m^	232 (32.3)	302 (34.3)	512 (33.2)

a Values are n (%).

b When asked what MNP are, mothers were considered correct if they indicated that it was a sachet of vitamins and minerals or that it was something that should be added to a child's food.

Identified benefits of MNP include improvements in appetite, energy, mental development, growth, immunity, health ,or strength.

c Only children at least 12 months of age were eligible to received MNP more than once. *n* = 943 for Kapilvastu and *n* = 815 for Achham.

d *n* = 634 in Kapilvastu and *n* = 709 in Achham.

e Analyses only in mothers who received MNP for the child (*n* = 731 in Kapilvastu and *n* = 900 in Achham).

f 96% of mothers who received a quantity of MNP other than 60 sachets (two boxes) reported receiving 30 sachets (1 box).

g Calculation for consumption of ≥75% of sachets received in the last distribution excludes mothers who recently received MNP and did not have time to use 75% of their sachets if they fed their child one sachet per day as recommended (i.e., mothers who received 30 sachets within the last 23 days and mothers who received 60 sachets within the last 45 days). *n* = 648 in Kapilsvastu and *n* = 773 in Achham.

h *n* = 613 in Kapilvastu and *n* = 330 in Achham. Percentages do not add up to 100% because mothers were allowed to cite multiple reasons.

i Includes mothers who stated she did not know enough about MNP (*n* = 265), who did not believe her child needed MNP (*n* = 55) and who were concerned about side effects of MNP (*n* = 23).

j External barriers include citing family members not wanting the mother to get MNP and lack of accessibility or transportation to the FCHV or health

k *n* = 719 for Kapilvastu and *n* = 880 for Achham.

l Mothers were asked if they perceived a positive effect, and if so, they were asked to describe the effect(s). Positive effects included improvements in appetite (*n* = 235), energy (*n* = 159), immunity/health (*n* = 605), strength (*n* = 534), growth (*n* = 189), or mental development (*n* = 308).

m Mothers were asked if they perceived a negative effect, and if so, they were asked to describe the effect(s). Negative effects include black or loose stool (*n* = 367), constipation (*n* = 18), nausea (*n* = 225), and vomiting (*n* = 181).

In the multivariable model, significant demographic predictors of MNP coverage included living in Achham, being 12–17 or 18–23 months (compared with 6–11 months), and maternal secondary education (compared with no education; Table 3). Significant predictors of maternal knowledge of appropriate use included being from Achham, having an older child (compared with 6–11 months), ethnicity (upper castes compared with the Dalit caste), and being from a food secure household (compared with those from severely food insecure households). Among mothers who had received MNP once and had children aged 12–23 months, significant predictors of repeat coverage included living in Achham, being 18–23 months (versus 12–17 months), and maternal secondary education (compared with no education). None of the demographic characteristics (other than district) was significantly associated with high intake of MNP.

**Table 3:** Socio‐economic and demographic predictors of high maternal knowledge of appropriate MNP use, MNP coverage, repeat MNP coverage, and high MNP intake in Achham and Kaplivastu districts, Nepal

	**MNP coverage** ^**a**^ ***N* = 2,576**					**Knowledge of appropriate MNP use** ^**b**^ ***N* = 2,576**					**Repeat MNP coverage** ^**c**^ ***N* = 1,342**					**High intake of MNP** ^**d**^ ***N* = 1,422**				
	***N***	***n* (%)**	*χ* ^**2**^ ***P*** a	**Adjusted PR [95% CI]** b	***P*** b	***N***	***n* (%)**	*χ* ^**2**^ ***P*** a	**Adjusted PR [95 % CI]** b	***P*** b	***N***	***n* (%)**	*χ* ^**2**^ ***P*** a	**Adjusted PR [95% CI]** b	***P*** b	***N***	***n* (%)**	*χ* ^**2**^ ***P*** a	**Adjusted PR [95% CI]** b	***P*** b
District			<0.001					<0.001					<0.001					0.002		
Achham	1,232	899 (73.0)		1.20 [1.07, 1.34]	0.002	1,232	498 (40.4)		1.36 [1.08, 1.71]	0.01	709	382 (53.9)		1.36 [1.08, 1.71]	0.01	775	267 (34.5)		0.81 [0.66, 1.00]	0.046
Kapilvastu	1,344	730 (54.3)		Reference		1,344	400 (29.8)		Reference	‐	633	256 (40.4)		Reference	‐	647	274 (42.4)		Reference	‐
Child's age			<0.001					0.02					<0.001					0.11		
6–11 months	819	287 (35.0)		Reference	‐	819	254 (31.0)		Reference	‐	‐	‐		‐	‐	214	68 (31.8)		Reference	‐
12–17 months	966	719 (74.4)		2.13 [1.90, 2.40]	<0.001	966	347 (35.9)		1.15 [1.04, 1.29]	0.01	719	295 (41.0)		Reference	‐	640	246 (38.4)		1.13 [0.92, 1.40]	0.24
18–23 months	791	623 (78.8)		2.26 [2.00, 2.55]	<0.001	791	297 (37.6)		1.21 [1.07, 1.36]	0.003	623	343 (55.1)		1.34 [1.18, 1.52]	<0.001	568	227 (40.0)		1.19 [0.95, 1.49]	0.14
Child's sex			0.58					0.63					0.99					0.79		
Male	1,369	859 (62.8)		‐	‐	1,369	483 (35.3)		‐	‐	694	330 (47.6)		‐	‐	745	281 (37.7)		‐	‐
Female	1,207	770 (63.8)		‐	‐	1,207	415 (34.4)		‐	‐	648	308 (47.5)		‐	‐	677	260 (38.4)		‐	‐
Ethnicity			<0.001					<0.001					<0.001					0.007		
Upper caste	1,028	733 (71.3)		Reference	‐	1,028	443 (43.1)		Reference	‐	579	304 (52.5)		Reference	‐	631	235 (37.2)		Reference	‐
Dalit hill/Terai	599	392 (65.4)		1.01 [0.95, 1.07]	0.77	599	181 (30.2)		0.81 [0.69, 0.95]	0.01	329	160 (48.6)		1.04 [0.83, 1.31]	0.75	344	112 (32.6)		0.85 [0.69, 1.05]	0.14
Other	949	504 (53.1)		0.96 [0.85, 1.07]	0.42	949	274 (28.9)		0.90 [0.70, 1.14]	0.38	434	174 (40.1)		0.98 [0.84, 1.15]	0.80	447	194 (43.4)		0.94 [0.77, 1.15]	0.56
Maternal education			<0.001					<0.001					0.06							
No formal education	1,286	784 (61.0)		Reference	‐	1,286	397 (30.9)		Reference	‐	660	303 (45.9)		Reference	‐	694	256 (36.9)	0.15	Reference	‐
Primary education	431	253 (58.7)		0.98 [0.92, 1.05]	0.59	431	142 (33.0)		1.06 [0.92, 1.22]	0.42	213	92 (43.2)		0.96 [0.80, 1.16]	0.69	226	99 (43.8)		1.17 [0.96, 1.43]	0.11
≥Secondary education	859	592 (68.9)		1.06 [1.01, 1.11]	0.02	859	359 (41.8)		1.14 [0.98, 1.32]	0.09	469	243 (51.8)		1.12 [1.01, 1.25]	0.03	502	186 (37.1)		1.02 [0.86, 1.21]	0.82
Main source of income			0.07					0.01					0.24					0.46		
Agriculture	1,506	974 (64.7)		1.03 [0.98, 1.09]	0.25	1,506	554 (36.8)		1.05 [0.93, 1.19]	0.42	815	398 (48.8)		‐	‐	850	330 (38.8)		‐	‐
Other	1,070	655 (61.2)		Reference	‐	1,070	344 (32.2)		Reference	‐	527	240 (45.5)		‐	‐	572	211 (36.9)		‐	‐
Household food Security^c^			0.03					0.005					0.52					0.07		
Food secure	1,393	849 (61.0)		Reference	‐	1,393	503 (36.1)		Reference	‐	705	333 (47.2)		‐	‐	743	298 (40.1)		Reference	‐
Mild or moderate insecurity	706	469 (66.4)		1.03 [0.97, 1.08]	0.33	706	259 (36.7)		0.99 [0.88, 1.13]	0.92	382	176 (46.1)		‐	‐	406	155 (38.2)		1.00 [0.87, 1.16]	0.95
Severely food insecure	477	311 (65.2)		0.97 [0.91, 1.04]	0.38	477	136 (28.5)		0.76 [0.63, 0.90]	0.002	255	129 (50.6)		‐	‐	273	88 (32.2)		0.92 [0.75, 1.14]	0.47
Household assets Tertile^d^			0.34					0.01					0.03					0.28		
Tertile 1 (more assets)	917	595 (64.9)		‐	‐	917	353 (38.5)		1.10 [0.96, 1.26]	0.15	489	211 (43.2)		0.87 [0.74, 1.01]	0.06	518	211 (40.7)		‐	‐
Tertile 2	985	607 (61.6)		‐	‐	985	332 (33.7)		1.04 [0.90, 1.20]	0.62	498	242 (48.6)		0.99 [0.86, 1.15]	0.93	533	193 (36.2)		‐	‐
Tertile 3 (fewer assets)	674	427 (63.4)		‐	‐	674	213 (31.6)		Reference	‐	355	185 (52.1)		Reference	‐	371	137 (36.9)		‐	‐

MNP coverage is defined as maternal report that she has received MNP for her child at least once.

Knowledge of appropriate MNP use defined as mother knowing the following seven points: (a) MNP should be introduced at 6 months; (b) MNP is no longer necessary after 23 months; (c) one sachet per day is the recommended dose; (d) MNP should be added to a small portion of food that the child can eat in one feeding; (e) MNP should not be added to hot food; (f) MNP should not be added to liquids; (g) food with MNP should be consumed within 30 min of adding MNP.

Repeat coverage defined as mothers of children 12–23 months who received MNP ≥2 times (among mothers who had received MNP at least once). Mothers of infants 6–11 months were removed from analyses because they were only eligible to receive MNP once.

High intake of MNP defined as consumption of ≥75% of sachets received in the last distribution excluding mothers who recently received MNP and did not have time to use 75% of their sachets if they fed their child one sachet per day as recommended (i.e., mothers who received 30 sachets within the last 23 days and mothers who received 60 sachets within the last 45 days).

a *p* values obtained from chi‐squared tests.

b Adjusted prevalence ratios (Adj. PR) and corresponding 95% confidence intervals (95% CI) and *p* values were obtained from generalized estimating equations with the log link and binomial distribution accounting for correlated errors using an exchangeable correlation structure. In order to develop parsimonious models, only variables that were significant at the *p* > 0.2 level in the chi‐squared tests were included in the multivariable models.

c Household food insecurity levels defined based on Household Food Insecurity Access Scale (Coates et al., 2007).

d Asset tertile determined from principle component analysis that included questions on whether each household had a radio, television, mobile phone, refrigerator, table, chair, bed, sofa, watch, computer, fan, traditional grain miller, bicycle, and electricity.

Mothers who spent more than 20 min travelling to see their FCHV were less likely to have received MNP than mothers who travelled 20 min or less (APR, 95% CI: 0.86 [0.78, 0.95] *p* = 0.002); however, there was no association with travel time to visit the FCHV and knowledge of appropriate use, repeat coverage, or high intake (Table 4). There was also no association with time spent travelling to a health centre and any of the outcomes of interest. Notably, mothers who had never sought help for her child from a health centre and mothers missing a value for travel time to their FCHV (mothers who had never interacted with their FCHV or received home visits only) were less likely to have received MNP and to have knowledge of appropriate MNP use compared with mothers who travelled less than 30 min to her health facility and less than 20 min to her FCHV, respectively.

**Table 4:** Programme indicators as predictors of MNP coverage, maternal knowledge of appropriate MNP use, repeat MNP coverage, and high intake of MNP in Achham and Kaplivastu districts, Nepal

	**MNP coverage** a ***N* = 2576**				**Knowledge of appropriate MNP use** b ***N* = 2576**				**Repeat MNP coverage** c ***N* = 1342**				**High intake of MNP** d ***N* = 1422**			
	***N***	***n* (%)**	**Adj. PR [95% CI]** e	***P***	***N***	***n* (%)**	**Adj. PR [95% CI]** e	***P***	***N***	***n* (%)**	**Adj. PR [95% CI]** e	***P***	***N***	***n* (%)**	**Adj. PR [95% CI]** e	**P**
**Among all mothers**																
Time spent travelling to nearest government health facility																
<30 min	877	596 (68.0)	Reference	‐	877	317 (36.2)	Reference	‐	705	359 (50.9)	Reference	‐	753	292 (38.8)	Reference	‐
≥30 min	1,351	870 (64.4)	1.00 [0.94, 1.06]	0.91	1,351	511 (37.8)	1.00 [0.89, 1.13]	0.97	498	232 (46.6)	0.90 [0.79, 1.03]	0.14	522	202 (38.7)	1.01 [0.87, 1.16]	0.92
Has never been	348	163 (46.8)	0.85 [0.78, 0.93]	<0.001	348	70 (20.1)	0.61 [0.48, 0.78]	<0.001	139	47 (33.8)	0.79 [0.61, 1.01]	0.06	147	47 (32.0)	0.78 [0.58, 1.06]	0.11
Time spent travelling to FCHV^f^																
<20 min	1,547	1,069 (69.1)	Reference*	‐	1,547	618 (40.0)	Reference	‐	887	435 (49.0)	Reference	‐	930	366 (39.4)	Reference	‐
≥20 min	350	220 (62.9)	0.86 [0.78, 0.95]	0.002	350	128 (36.6)	0.93 [0.80, 1.07]	0.30	178	93 (52.3)	1.07 [0.90, 1.25]	0.45	196	65 (33.2)	0.92 [0.74, 1.15]	0.46
Missing^g^	679	340 (50.1)	0.81 [0.74, 0.89]	<0.001	679	152 (22.4)	0.66 [0.56, 0.78]	<0.001	277	110 (39.7)	0.85 [0.74, 0.98]	0.03	296	110 (37.2)	0.98 [0.82, 1.18]	0.84
Mother has heard an MNP radio message^h^																
Yes	349	349 (100.0)	1.07 [1.02, 1.13]	0.006	349	178 (51.0)	1.02 [0.92, 1.15]	0.68	277	163 (58.8)	1.19 [1.07, 1.32]	0.001	291	117 (40.2)	1.03 [0.90, 1.18]	0.68
No	2,227	1,280 (57.5)	Reference*	‐	2,227	720 (32.3)	Reference	‐	1,065	475 (44.6)	Reference	‐	1,131	424 (37.5)	Reference	‐
Mother received IYCF‐MNP counselling from a health worker (HW)^i^																
Yes	913	911 (99.8)	1.35 [1.26, 1.45]	<0.001	913	443 (48.5)	1.19 [1.05, 1.34]	0.01	731	406 (55.5)	1.30 [1.13, 1.50]	<0.001	778	297 (38.2)	1.07 [0.93, 1.23]	0.35
No	1,663	718 (43.2)	Reference*	‐	1,663	455 (27.4)	Reference	‐	611	232 (38.0)	Reference	‐	644	244 (37.9)	Reference	‐
Mother received IYCF‐MNP counselling from FCHV^j^																
Yes	1,312	1,309 (99.8)	2.97 [2.55, 3.47]	<0.001	1,312	630 (48.0)	1.99 [1.72, 2.31]	<0.001	1,078	545 (50.6)	1.28 [1.09, 1.50]	0.003	1,143	457 (40.0)	1.39 [1.12, 1.72]	0.003
No	1,264	320 (25.3)	Reference*	‐	1,264	268 (21.2)	Reference	‐	264	93 (35.2)	Reference	‐	279	84 (30.1)	Reference	‐
Type of FCHV IYCF‐MNP counselling^j^:																
Group only	287	286 (99.7)	3.03 [2.61, 3.53]	<0.001	287	118 (41.1)	1.94 [1.61, 2.33]	<0.001	237	113 (47.7)	1.26 [1.04, 1.53]	0.02	253	112 (44.3)	1.60 [1.27, 2.01]	<0.001
Individual only	312	311 (99.7)	3.05 [2.61, 3.57]	<0.001	312	154 (49.4)	2.04 [1.71, 2.43]	<0.001	261	98 (37.6)	1.06 [0.85, 1.33]	0.60	281	100 (35.6)	1.20 [0.94, 1.54]	0.15
Individual and group	713	712 (99.9)	2.87 [2.45, 3.36]	<0.001	713	358 (50.2)	1.98 [1.68, 2.35]	<0.001	580	334 (57.6)	1.42 [1.20, 1.68]	<0.001	609	245 (40.2)	1.43 [1.13, 1.80]	0.003
No counselling	1,264	320 (25.3)	Reference*	‐	1,264	268 (21.2)	Reference	‐	264	93 (35.2)	Reference*	‐	279	84 (30.1)	Reference	‐
Received reminder to pick‐up MNP from FCHV or HW^k^																
Yes	1,064	1,062 (99.8)	1.38 [1.29, 1.47]	<0.001	‐	‐	‐	‐	1,071	537 (50.1)	1.07 [0.94, 1.23]	0.29	‐	‐	‐	‐
No	1,512	567 (37.5)	Reference*	‐	‐	‐	‐	‐	271	101 (37.3)	Reference	‐	‐	‐	‐	‐
**Among mothers who received FCHV counselling** l																
Frequency of mother‐FCHV interactions^j^																
<Once per month	317	317 (100.0)	Reference*	‐	317	147 (46.4)	Reference	‐	275	124 (45.1)	Reference*	‐	280	113 (40.4)	Reference	‐
Monthly	401	400 (99.8)	1.00 [0.99, 1.00]	0.54	401	178 (44.4)	0.90 [0.77, 1.06]	0.20	331	170 (51.4)	1.14 [0.97, 1.35]	0.12	347	132 (38.0)	1.01 [0.84, 1.22]	0.92
>Once per month	594	592 (99.7)	1.00 [0.99, 1.00]	0.18	594	305 (51.4)	1.01 [0.87, 1.18]	0.87	472	251 (53.2)	1.09 [0.92, 1.30]	0.30	516	212 (41.1)	1.09 [0.90, 1.32]	0.39
Satisfaction with FCHV services^j^																
Very satisfied	293	293 (100.0)	1.00 [1.00, 1.00]	0.09	293	165 (56.3)	1.17 [1.02, 1.33]	0.02	245	129 (52.7)	0.99 [0.85, 1.14]	0.85	252	119 (47.2)	1.16 [0.98, 1.39]	0.09
Other response	1019	1016 (99.7)	Reference*	‐	1019	465 (45.6)	Reference	‐	833	416 (49.9)	Reference	‐	891	338 (37.9)	Reference	‐
**Among mothers who received HW counselling** m																
Frequency of health centre visits for child^i^																
<once per month	328	328 (100.0)	Reference*	‐	328	149 (45.4)	Reference	‐	279	160 (57.4)	Reference	‐	285	107 (37.5)	Reference	‐
Monthly	289	288 (99.7)	1.00 [1.00, 1.00]	0.37	289	145 (50.2)	1.03 [0.89, 1.20]	0.67	227	123 (54.2)	0.96 [0.83, 1.12]	0.63	243	92 (37.9)	0.96 [0.84, 1.10]	0.54
>once per month	296	295 (99.7)	1.00 [1.00, 1.00]	0.41	296	149 (50.3)	1.11 [0.95, 1.29]	0.18	225	123 (54.7)	0.94 [0.81, 1.08]	0.38	250	98 (39.2)	0.97 [0.78, 1.21]	0.80
Satisfaction with health services^i^																
Very satisfied	168	168 (100.0)	1.00 [1.00, 1.01]	0.16	168	85 (50.1)	1.04 [0.88, 1.23]	0.67	140	83 (59.3)	1.03 [0.90, 1.17]	0.68	150	61 (40.7)	1.06 [0.89, 1.27]	0.49
Other response	745	743 (99.7)	Reference*	‐	745	358 (48.1)	Reference	‐	591	323 (54.7)	Reference	‐	628	236 (37.6)	Reference	‐

a MNP coverage is defined as aternal report that she has received MNP for her child at least once.

b Knowledge of appropriate MNP use defined as mother knowing the following seven points: (a) MNP should be introduced at 6 months; (b) MNP is no longer necessary after 23 months; (c) one sachet per day is the recommended dose; (d) MNP should be added to a small portion of food that the child can eat in one feeding; (e) MNP should not be added to hot food; (f) MNP should not be added to liquids; (g) Food with MNP should be consumed within 30 min of adding MNP.

c Repeat coverage defined as mothers of children 12–23 months who received MNP ≥2 times (among mothers who had received MNP at least once). Mothers of infants 6–11 months were removed from analyses because they were only eligible to receive MNP once.

d High intake of MNP defined as consumption of ≥75% of sachets received in the last distribution excluding mothers who recently received MNP and did not have time to use 75% of their sachets if they fed their child one sachet per day as recommended (i.e., mothers who received 30 sachets within the last 23 days and mothers who received 60 sachets within the last 45 days).

e Adjusted prevalence ratios (Adj PR) and corresponding 95% confidence intervals (95% CI) and *p* values were obtained from generalized estimating equations with the log link and binomial distribution accounting for correlated errors using an exchangeable correlation structure. When the log‐binomial model did not converge, a Poisson distribution, which gives a consistent but less efficient estimate (Zou, 2004) was used. Log‐poisson models are indicated with an asterisk (*) after the reference term. Multivariable models adjust for all sociodemographic covariates that were significant at the *p* < 0.2 level in chi‐square tests for the outcome of interest. For coverage, this includes district, child's age, ethnicity, maternal education, source of household income, and household food insecurity level. For knowledge: district, child's age, ethnicity, maternal education, source of household income, household food insecurity level, and household asset tertile. For repeat coverage: district, child's age, ethnicity, maternal education and household asset tertile. For high intake: district, child's age, ethnicity, maternal education and household food security level.

f Model also adjusts for the amount of time it takes the mother to travel to her health centre.

g Mothers missing data on travel time to their FCHV include: mothers who have never interacted with their FCHV (*n* = 228), mothers who receive home visits only (*n* = 449) and other (*n* = 2) in full sample of children 6–23 months.

h Multivariable models with MNP radio message exposure as a predictor also adjust for whether the mother ever received IYCF‐MNP counselling from a FCHV and/or health worker.

i Multivariable models with health worker indicators as predictors also adjust for whether the mother received IYCF‐MNP counselling from a FCHV and whether she ever heard an MNP radio message.

j Multivariable models with FCHV indicators as predictors also adjust for whether the mother received IYCF‐MNP counselling from a health worker and whether she ever heard an MNP radio message.

k Model for receipt of a reminder from a health worker or FCHV also adjusts for whether the mother received counselling from a health worker or FCHV, and whether she heard an MNP radio message.

l Only among mothers who had received MNP counselling from an FCHV (*n* = 1,312 in full sample of children 6–23 months).

m Only among mothers who had received MNP counselling from a HW (*n* = 913 in full sample of children 6–23 months).

Compared with those who did not receive IYCF‐MNP counselling from an FCHV, mothers who received FCHV IYCF‐MNP counselling were significantly more likely to receive MNP sachets at least once (APR, 95% CI: 2.97 [2.55, 3.47], *p* < 0.001), correctly answer all seven questions on appropriate use (APR, 95% CI: 1.99 [1.72, 2.31], *p* < 0.001), receive their second MNP distribution if they had received their first distribution and had a child who was 12–23 months (APR, 95% CI: 1.28 [1.09, 1.50], *p* = 0.003), and to feed their child ≥75% of sachets received (APR, 95% CI: 1.39 [1.12, 1.72], *p* = 0.003). Counselling from a health worker was also significantly associated with coverage (APR, 95% CI: 1.35 [1.26, 1.45], *p* = <0.001), knowledge on appropriate use (APR, 95% CI: 1.19 [1.05, 1.34], *p* = 0.01), and repeat coverage (APR, 95% CI: 1.30 [1.13, 1.50], *p* < 0.001) but not high intake. Hearing an MNP radio messages was associated with the coverage indicators only (APR, 95% CI: 1.07 [1.02, 1.13], *p* = 0.006 for coverage and 1.19 [1.07, 1.32], *p* = 0.001 for repeat coverage). Mothers who received a reminder to pick‐up MNP from either a health worker or an FCHV were also more likely to receive MNP than mothers who never received a reminder.

As for type of FCHV counselling, mothers who received individual, group, or both types of IYCF‐MNP counselling were all significantly more likely to receive MNP at least once and to correctly answer all seven appropriate use questions compared with mothers who did not receive FCHV IYCF‐MNP counselling. Mothers who received FCHV group counselling or group‐plus‐individual counselling were also significantly more likely to receive MNP repeatedly (when age appropriate) and to feed their children ≥75% of the MNP compared with mothers who did not receive IYCF‐MNP counselling from an FCHV; however, we did not find a significant difference in prevalence of repeat coverage or high intake when comparing mothers who received individual counselling only and mothers who did not receive FCHV IYCF‐MNP counselling. Notably, the reference group (mothers who did not receive IYCF‐MNP counselling from an FCHV) changed substantially for the repeat coverage (*n* = 264) and high intake (*n* = 279) analyses compared with the knowledge and coverage analyses (*n* = 1264), because the majority of mothers who did not receive FCHV counselling also did not receive MNP and were thus excluded from these analyses. Among mothers who received IYCF‐MNP counselling from their FCHV, we did not find that the usual frequency of mother–FCHV interactions for the child was associated with knowledge, coverage, repeat coverage, or high intake; being “very satisfied” with her FCHV was, however, positively associated with maternal knowledge of appropriate use. We did not find an association between health centre visit frequency or maternal satisfaction with any of the MNP indicators.

Among mothers who had tried feeding their child MNP, their perceptions of MNP were strongly associated with repeat coverage and high intake (Table 5). Among mothers of children aged 12–23 months who tried MNP, mothers who thought the child liked food with MNP were 1.43 (95% CI [1.27, 1.60]) times more likely to receive MNP a second time, and among mothers of children aged 6–23 months, those who thought their child liked food with MNP were 5.24 (95% CI [4.21, 6.53]) times more likely to feed their child at least 75% of their last MNP distribution compared with mothers who did not think their child liked food with MNP. By contrast, mothers who reported their child seemed disturbed by changes in the colour, taste, or smell of food due to MNP were 18% less likely to pick‐up MNP a second time (APR, 95% CI: 0.83 [0.76, 0.92], *p* < 0.001), and less than half as likely to feed their child 75% of the received MNP (APR, 95% CI: 0.40 [0.34, 0.48, *p* < 0.001), compared with mothers who did not identify organoleptic changes or did not think their child was disturbed by the changes. Finally, mothers who perceived a positive effect of MNP on their child were 1.51 (95% CI [1.32, 1.72]) times more likely to pick‐up MNP a second time and 3.36 (95% CI [2.80, 4.03]) times more likely to feed their child at least three quarters of the MNP they received, whereas mothers who perceived a negative effect were 12% less likely to pick‐up MNP a second time (APR, 95% CI: 0.88 [0.79, 0.98], *p* = 0.02), and less than half as likely as those who did not perceive any negative effects to feed their child ≥75% of the MNP they received (APR, 95% CI: 0.49 [0.40, 0.59], *p* < 0.001).

**Table 5:** Maternal experience with and perceptions of MNP as predictors of repeat coverage and high MNP intake in Achham and Kaplivastu districts, Nepal

	**Repeat MNP coverage** a ***N* = 1316**				**High intake of MNP** b ***N* = 1394**			
	***N***	***n* (%)**	**Adj. PR [95% CI]** c	***P***	***N***	***n* (%)**	**Adjusted PR [95% CI]** c	**P**
Mother reports that the child likes to eat food with MNP								
Yes	664	377 (56.8)	1.43 [1.27, 1.60]	<0.001	696	457 (65.7)	5.24 [4.21, 6.53]	<0.001
No	652	256 (39.3)	Reference	‐	698	84 (12.0)	Reference	‐
Mother reports that adding MNP changed the colour of the food								
Yes	453	215 (47.5)	0.96 [0.85, 1.08]	0.47	487	182 (37.4)	0.96 [0.84, 1.11]	0.88
No	863	418 (48.4)	Reference	‐	907	359 (39.6)	Reference	‐
Mother reports that adding MNP changed the taste of the food								
Yes	634	279 (44.0)	0.86 [0.76, 0.96]	0.01	678	171 (25.2)	0.50 [0.43, 0.59]	<0.001
No	682	354 (51.9)	Reference	‐	716	370 (51.7)	Reference	‐
Mother reports that adding MNP changed the smell of the food								
Yes	353	168 (47.6)	0.95 [0.85, 1.06]	0.36	375	101 (26.9)	0.65 [0.54, 0.77]	<0.001
No	963	465 (48.3)	Reference	‐	1019	440 (43.2)	Reference	‐
Mother reports that the child disliked the change in colour, taste or smell of the food								
Yes	652	282 (43.3)	0.83 [0.76, 0.92]	<0.001	702	153 (21.8)	0.40 [0.34, 0.48]	<0.001
No	664	351 (52.9)	Reference	‐	692	388 (56.1)	Reference	‐
Mother perceived a positive effect of MNP^d^								
Yes	673	397 (59.0)	1.51 [1.32, 1.72]	<0.001	711	423 (59.5)	3.36 [2.80, 4.03]	<0.001
No	643	236 (36.7)	Reference	‐	683	118 (17.3)	Reference	‐
Mother perceived a negative effect of MNP^e^								
Yes	433	187 (43.2)	0.88 [0.79, 0.98]	0.02	476	104 (21.9)	0.49 [0.40, 0.59]	<0.001
No	883	446 (50.5)	Reference	‐	918	437 (47.6)	Reference	‐

a Repeat MNP coverage defined as mothers of children 12–23 months who received MNP at least two times (among mothers who had received MNP at least once). Mothers of infants 6–11 months were removed from analyses because they were only eligible to receive MNP once. Analyses are conducted only among mothers who had tried feeding their child MNP.

b High intake of MNP is defined as consumption of ≥75% of sachets received from last distribution. Analysis excludes mothers who did not receive MNP and also mothers who received MNP recently and did not have sufficient time to feed the child one sachet per day and still use 75% of sachets (i.e., those who received 30 sachets within the last 23 days and those who received 60 sachets within the last 45 days). Analysis is conducted only among mothers who had tried feeding their child MNP.

c Adjusted prevalence ratios (Adj PR) and corresponding 95% confidence intervals (95% CI) and *p* values were obtained from generalized estimating equations with the log link and binomial distribution accounting for correlated errors using an exchangeable correlation structure. Multivariable models adjust for all sociodemographic covariates that were significant at the *p* < 0.2 level in chi‐square tests for the outcome of interest. For repeat coverage, this includes district, child's age, ethnicity, maternal education and household asset tertile; for high intake: district, child's age, ethnicity, maternal education and household food security level. All models also adjust for whether the mother ever heard an MNP radio message and whether she received IYCF‐MNP counselling from an FCHV or health worker.

d Positive effects of MNP include improvements in health, growth, immunity, appetite, energy/activity, or mental development.

e Negative effects of MNP include black or loose stool, constipation, vomiting, or nausea.

## 4 DISCUSSION

In this survey, that was representative of children aged 6–23 months in two districts in Nepal that were part of a post‐pilot, scale‐up of an integrated IYCF‐MNP programme, we found that approximately two thirds of targeted mothers had received MNP for their child, but only one third of mothers of children aged 12–23 months (who were old enough to receive a second distribution) had received MNP sachets at least two times. The coverage indicators from this survey are lower than those from the Nepal IYCF‐MNP pilot programme (Jefferds et al., 2015), potentially reflecting the reduction in resources per district as the programme scaled and implementation was fully integrated into the national health system without additional partner supports. A recent review found that countries implementing free health sector MNP distribution programmes have reported coverage (defined by each intervention) ranging from 32% to 83% (Jefferds et al., 2015; Korenromp et al., 2016; Reerink et al., 2017); however, only two studies reported indicators for programmes implemented at scale (as opposed to pilot programmes; CDC & UNICEF, 2013; Olney, Rawat, & Ruel, 2011).

It has been proposed that >70% should be considered the coverage target for MNP programmes (Reerink et al., 2017). This threshold is lower than the 90% coverage target for biannual vitamin A supplementation due to the greater behaviour change required by regular MNP consumption but also estimated to be sufficient to achieve nutritional impact (Reerink et al., 2017). Our findings emphasize the need to strengthen programme coverage in Nepal, and likely in other countries, from both the supply and demand sides. Notably, we observed important disparities in our programme indicators by maternal education, ethnicity, household food insecurity, and distance mothers lived from their FCHV. These findings highlight the importance of investing in programme equity, particularly as IYCF‐MNP programmes scale and become part of national and subnational programmes, to ensure that *all* mothers, including those of lower education levels and those from food insecure households have access to MNP information, reminders, and counselling. Our findings also highlight that simplified, targeted messages for vulnerable community members may also contribute to greater MNP knowledge and appropriate use (Monterrosa et al., 2013; Sanghvi, Jimerson, Hajeebhoy, Zewale, & Nguyen, 2013). Notably, among the mothers who received MNP, over 80% had received it in the last 6 months (as recommended); however, the majority received only one box instead of the recommended two. In addition, among mothers who reported never getting MNP for their child, one in five said it was due to stock out, which further limits MNP coverage and may also undermine MNP demand. Thus, investments in IYCF‐MNP behaviour change communication should be complemented with investments in the supply chain to ensure adequate MNP stock at the facility and community level, as well as capacity building for frontline workers to ensure they understand the recommended distribution quantity and how to trouble‐shoot supply chain challenges.

Once mothers receive MNP, intake adherence can be defined as the initiation, appropriate use, and continued use of MNP (Tumilowicz et al., 2017). In the Nepal IYCF‐MNP programme, 97.9% of mothers who received MNP initiated use and the majority of all mothers were able to correctly answer each MNP appropriate use question. However, only one third of mothers were able to answer all seven appropriate‐use questions correctly, and only 38.9% of children whose mothers received MNP were classified as having “high intake.” Interestingly, we found that FCHV group counselling or group‐plus‐individual counselling were both significantly associated with repeat coverage and high intake of MNP, but individual FCHV counselling was not. We also did not find an association between hearing the MNP radio messages or receiving health worker IYCF counselling and high intake. The Stages of Change theory (Prochaska et al., 1994) highlights that mass media may be important in the early phases of behaviour change; however skills building, social support, and continued positive reinforcement are essential for maintaining changes in health behaviour. In Nepal, hearing the MNP radio messages likely served as a cue to action to remind mothers to pick‐up MNP, as demonstrated by the association between hearing the radio messages and coverage indicators; however, the brevity of the radio messages was likely insufficient to support mothers in establishing and maintaining a daily routine for feeding their child MNP. A recent systematic review of nutrition education and mass media interventions found that of the 18 studies identified, only three directly assessed the impact of mass media alone, and none showed significant changes in behaviour; however, when mass media was combined with individual or group education, several interventions were able to improve IYCF practices (Graziose, Downs, O'Brien, & Fanzo, 2018). Other studies have also documented the importance of group counselling for improving IYCF practices generally (Flax et al., 2014; Nguyen et al., 2014; White et al., 2016), and one study from Peru showed that the positive encouragement from neighbours and relatives encouraged mothers to use MNPs (Creed‐Kanashiro, Bartolini, Abad, & Arevalo, 2016). Individual counselling and home visits are likely important for marginalized mothers who are unlikely to attend group meetings and, as shown in this evaluation in Nepal, can contribute to improved MNP knowledge and coverage; however, the added peer support of group counselling may be particularly important for maintaining motivation and resolving barriers that are necessary for sustained changes in behaviour.

Despite the fact that both facility‐based and community‐based health workers received IYCF‐MNP training and both were expected to distribute MNP, over 80% of mothers received MNP from their FCHV, and the receipt of FCHV counselling had a consistently stronger association with knowledge of appropriate use, coverage, and high intake than health worker counselling. This is consistent with the Nepal pilot programme findings which showed that the two districts that used an FCHV delivery model had higher coverage and repeat coverage than the districts which used a health facility‐based distribution model only (83% vs. 52%; Jefferds et al., 2015). FCHVs are women (usually mothers) who live in local communities and are often active leaders in community social networks. FCHVs often interact with mothers more frequently and for longer durations than facility‐based health workers and are thus better positioned to deliver consistent IYCF‐MNP counselling and to help mothers troubleshoot as challenges arise. Other studies have also shown that community health workers are a viable and reliable channel for the delivery of MNP and IYCF‐MNP counselling (Locks et al., 2017; Olney et al., 2011). The reiteration of key messages through facility‐based health workers can complement community‐based counselling; however, our findings highlight that community health worker counselling may be especially important for supporting a deep understanding of how to use MNP and for supporting sustained use.

If MNPs are well‐received by the community, they could also contribute to community recognition of the FCHV and ultimately improve motivation; a recent systematic review of CHW performance found that CHWs who provided curative services or physical goods reported greater recognition from their community and ultimately more motivation (Kok et al., 2015). In the Nepal pilot programme, however, 15 months into programme implementation, 57% and 44% of FCHVs in the FCHV‐delivery districts and health facility‐delivery districts, respectively, reported needing more support or disliking the added work of MNP (Jefferds et al., 2015). We did not assess FCHV motivation or capacity in this evaluation, but in a separate analysis from this survey population, we did find that mothers who received MNP from their FCHV were more likely to report being “very satisfied” with the performance of their FCHV (Locks et al., 2018), potentially due to improved motivation and quality of counselling. Future research and monitoring at the CHW level are essential to understand how the introduction of MNP affects CHW motivation and performance, as well as how this changes with the provision of adequate supervision and support, and also over time as programmes mature (Vossenaar et al., 2017).

In this analysis, we found that almost half of mothers who fed their child MNP reported that their child disliked the food with MNP due to changes in the taste, smell, or colour of food. We also found that maternal perceptions of a lack of organoleptic (taste, smell, and colour) changes of food when MNP was added, child's acceptance of foods with MNP, and perceived positive changes in the child due to MNP were strong predictors of high MNP intake. The frequency of maternal report of organoleptic changes may be a sign of inappropriate preparation of foods with MNP by mothers (such as adding MNP to hot foods, soups or other liquids, and/or very small portions of food; Tumilowicz et al., 2017); however, we did not find in this analysis that knowledge of appropriate use was associated with maternal report of organoleptic changes. Other MNP interventions have encountered product quality issues that resulted in organoleptic changes to food (Locks et al., 2017; Schauer et al., 2017). Taken together, these findings highlight the importance of not only ensuring a high quality MNP product (Schauer et al., 2017) but also incorporating behaviour change strategies that support mothers as they try different strategies to encourage the child to eat foods with MNP, such as mixing with highly flavourful foods such as bananas or curry, making sure the amount of food served to the child is sufficient to thoroughly mix in the MNP, or adding MNP to the child's food without their knowledge (Jefferds et al., 2010). Behaviour change interventions should also reiterate to mothers throughout the programme cycle which negative changes mothers might expect to see in their children due to MNP (such as changes in consistency and colour of stool) but also consistently emphasize the positive changes mothers might see as well, such as the increased strength and appetite reported by many Nepali mothers.

This analysis has several limitations. The cross‐sectional design prevents analysis of temporal trends and the observational study design prevents us from ruling out associations are due to residual confounding. However, notably, the analyses adjust for several important sociodemographic characteristics such as maternal education and household food security level. The analysis is also limited to surveys with mothers only and is unable to elucidate FCHV or health worker barriers and opportunities in programme implementation. We are also dependent on maternal recall for questions on coverage and intake, though a study from the Nepal pilot IYCF‐MNP programme found that maternal recall on the number of sachets consumed and the number of sachets observed were highly correlated (Ng'eno et al., 2017). This analysis also has several strengths. The survey was conducted in a sample that was representative of all children 6–23 months in Achham and Kapilvastu districts, thus allowing conclusions that are generalizable to a large programme area. In addition, the large sample provided ample power for multivariable models adjusting for several key covariates, and the use of log‐binomial as opposed to logistic regression models prevents the overestimation of the prevalence ratios given the relatively common outcomes (Spiegelman & Hertzmark, 2005).

## 5 CONCLUSIONS

Continued resources and support for MNP programmes as they scale to the national and subnational level during post‐pilot phase are essential to support programme coverage and the adoption and maintenance of positive behaviour change. In this study, we found that counselling by health workers and FCHVs, and hearing MNP radio messages, were all independently associated with MNP coverage and repeat coverage; however, counselling from an FCHV had the strongest association with maternal MNP knowledge, coverage, and high intake. Counselling by community health workers may play a vital role in improving the coverage of and adherence to MNP programmes in similar settings globally, though future research should address how to best utilize volunteer workers without over‐burdening their workload.

## CONFLICTS OF INTEREST

The findings and conclusions in this report are those of the authors and do not necessarily represent the official position of the Centers for Disease Control and Prevention, UNICEF, or the Government of Nepal. The authors declare that they have no conflicts of interest.

## CONTRIBUTIONS

MEJ, PD, ZM, DW, and NP designed the evaluation; PD, RP, SC, NP, NJ, and BL oversaw survey implementation; PD, RP, SC, NP, NJ, BL, MEJ, ZM, and DW provided training, supervision, and support of data collectors and interpretation and approval of analyses; LML analysed data; and LML, AG, and MEJ wrote the paper and had primary responsibility for final content. All authors read and approved the final manuscript.

## Supporting information

Table S1: Explanation of covariates included in multivariable models in Tables 4 & 5.
