# Supplementary material for: Predictors of micronutrient powder (MNP) knowledge, coverage, and consumption during the scale‐up of an integrated infant and young child feeding (IYCF‐MNP) programme in Nepal
Source: Matern Child Nutr. 2019 Oct 17;15(Suppl 5):e12712. doi: 10.1111/mcn.12712 (PMC6856851; doi:10.1111/mcn.12712)
Supplement: Supplementary file 1 — Table S1: Explanation of covariates included in multivariable models in Tables 4 & 5. [file MCN-15-e12712-s001.docx]

**Supplemental Table 1: Explanation of covariates included in multivariable models in Tables 4 & 5**

| **Program Indicator of Interest** | **Covariates adjusted for in the multivariable model to assess the relationship between that indicator of interest and the 4 MNP outcomes.** |
| --- | --- |
| **Distance Decay** |  |
| - Time spent travelling to the nearest health center (proxy for geographic isolation at the district level) | Sociodemographic (SES) characteristics only |
| - Time spent travelling to see the Female Community Health Volunteer (FCHV) (proxy for geographic isolation at the community level) | SES + travel time to nearest health center |
| **Exposure to MNP information** |  |
| - Mother heard MNP radio messages | Single model for all sources of information: SES + the other two sources of MNP information |
| - Mother received IYCF-MNP counselling from a health worker (HW) |  |
| - Mother received IYCF-MNP counselling from FCHV |  |
| **Detailed indicators on source of MNP information** |  |
| - Type of FCHV IYCF-MNP counselling (individual, group, none or both) | SES + radio exposure + HW MNP counselling |
| - Receipt of reminder to pick-up last MNP distribution from an FCHV or HW | SES + radio exposure + HW MNP counselling + FCHV counselling |
| **Assessment of FCHV-related indicators**  **(among those who received FCHV counselling)** |  |
| - Frequency of mother-FCHV interactions | SES + radio exposure + HW MNP counselling |
| - Maternal satisfaction with FCHV services | SES + radio exposure + HW MNP counselling |
| **Assessment of HW-related indicators**  **(among those who received HW counselling)** |  |
| - Frequency of mother-HW interactions | SES + radio exposure + FCHV MNP counselling |
| - Maternal satisfaction with HW services | SES + radio exposure + FCHV MNP counselling |
| **Maternal Perceptions of MNP** |  |
| - Mother reports the child likes to eat food with MNP | SES + radio exposure + HW MNP counselling + FCHV counselling |
| - Mother perceived that MNP changed the color, taste or smell of food | SES + radio exposure + HW MNP counselling + FCHV counselling |
| - Mother perceived that the child was bothered by the change in color, taste or smell of food | SES + radio exposure + HW MNP counselling + FCHV counselling |
| - Mother perceived positive effects of MNP | SES + radio exposure + HW MNP counselling + FCHV counselling |
| - Mother perceived negative effects of MNP | SES + radio exposure + HW MNP counselling + FCHV counselling |
